# Immune Checkpoint Inhibitors and Long-term Survival of Patients With Metastatic Urothelial Cancer

**DOI:** 10.1001/jamanetworkopen.2023.7444

**Published:** 2023-04-12

**Authors:** Alec Zhu, Jorge Alberto Garcia, Bishoy Faltas, Petros Grivas, Pedro Barata, Jonathan E. Shoag

**Affiliations:** 1 Department of Urology, NewYork-Presbyterian Hospital, Weill Cornell Medicine, New York, New York; 2 Department of Medicine, University Hospitals Cleveland Medical Center, Case Western Reserve University School of Medicine, Cleveland, Ohio; 3 Division of Hematology and Medical Oncology, Department of Medicine, NewYork-Presbyterian Hospital, Weill Cornell Medicine, New York, New York; 4 Fred Hutchinson Cancer Center, Division of Medical Oncology, Department of Medicine, University of Washington Medicine, Seattle; 5 Department of Urology, University Hospitals Cleveland Medical Center, Case Western Reserve University School of Medicine, Cleveland, Ohio

## Abstract

This cohort study uses published clinical trial data to assess long-term survival of patients with metastatic urothelial carcinoma who are treated with immune checkpoint inhibitors.

## Introduction

Treatment of metastatic urothelial carcinoma (mUC) has shifted with the introduction of immune checkpoint inhibitors (ICIs). Platinum-based combination chemotherapy has been the mainstay first-line induction treatment of mUC for eligible patients. However, up to 50% of patients with advanced UC may not be candidates for cisplatin-based chemotherapy due to comorbidities, while others may be able to receive carboplatin-based chemotherapy.^1^ The use of ICIs within this treatment population has increased in recent years, with the JAVELIN Bladder 100 trial demonstrating overall survival (OS) and progression-free survival (PFS) benefits with avelumab for patients who achieve stable disease or response with chemotherapy.^2^ Although ICIs achieved long-term durable responses and OS with other tumor types (eg, melanoma), their ability to achieve similar results with mUC is not as well defined.^3^ We compiled published clinical trial data on the long-term OS and PFS results of patients with mUC treated with ICIs to understand whether there is support for ICIs providing long-term survival benefit.

## Methods

We identified 6 clinical trials evaluating pembrolizumab, avelumab, and nivolumab in the first-line, maintenance, and salvage settings for mUC; only treatments that received and have ongoing approvals from the US Food and Drug Administration (FDA) were included. Published articles of completed clinical trials were reviewed to determine the median age, sex, follow-up time, OS, PFS, objective response rates, and complete response rates within the treatment groups.^4^ The numbers at risk, which signify the most reliable indicator of long-term survival, among the treated cohorts within each clinical trial were tabulated at baseline and 12, 24, 36, and 48 months of follow-up. This study followed the Strengthening the Reporting of Observational Studies in Epidemiology (STROBE) reporting guideline. University Hospital’s institutional review board certified that this study did not constitute human participants research.

## Results

The median patient age ranged from 66 to 74 years, and the proportion of men ranged from 71.5% to 78% across included trials. The numbers at risk within the treatment groups of each trial for OS and PFS outcomes are shown in the Figure and the median OS or PFS and response rates are shown in the Table. Of 1021, 344, and 782 patients treated with first-line, maintenance, or salvage ICIs, respectively, no patients had continued follow-up and were alive or progression-free at 48 months.

**Figure.**
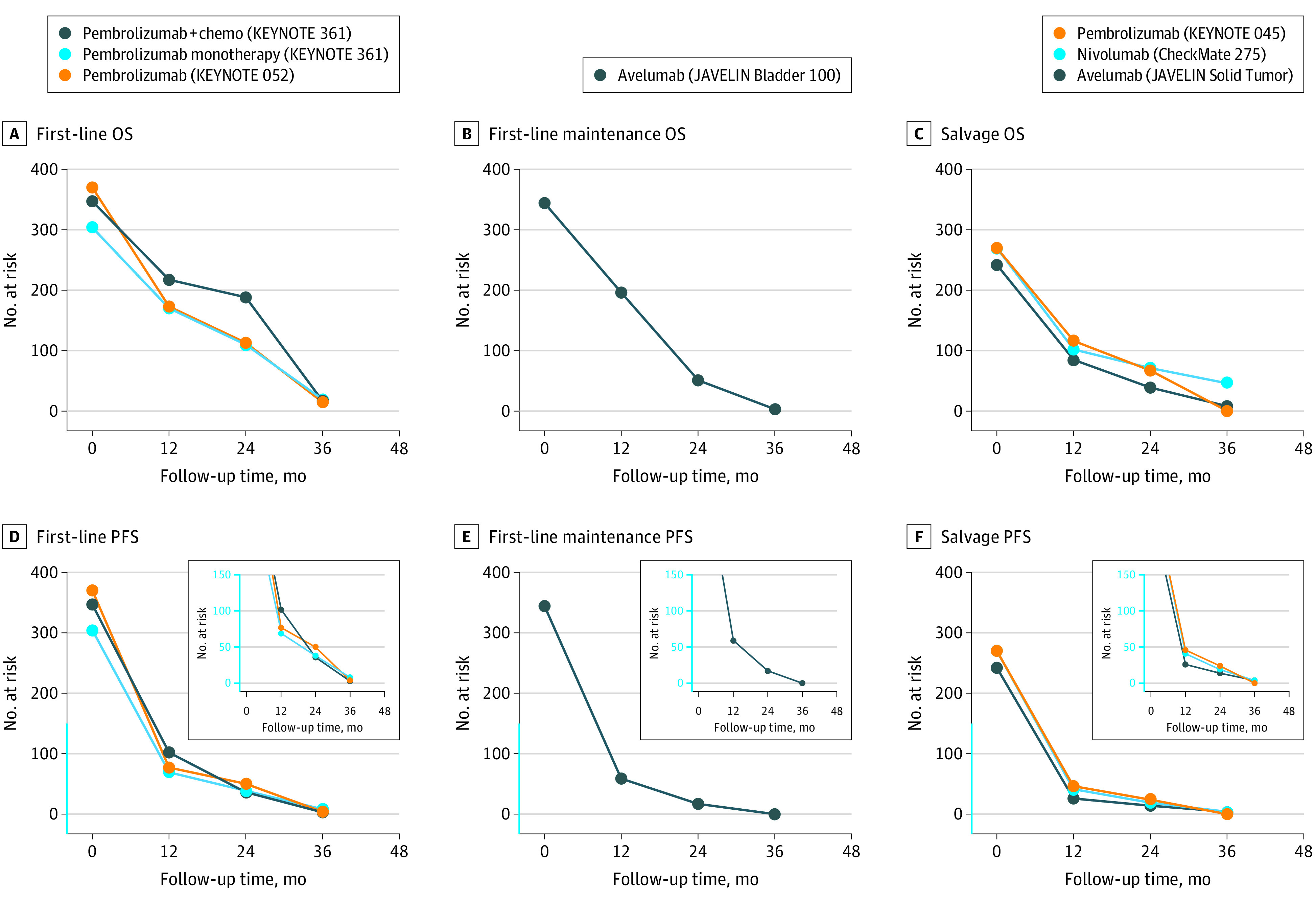
Overall Survival (OS) and Progression-Free Survival (PFS) in Trials of Immune Checkpoint Inhibitors for Metastatic Urothelial Cancer The numbers at risk within the treatment group of each clinical trial were recorded based on follow-up time for OS and PFS outcomes for first-line treatment, first-line maintenance treatment, and salvage treatment.

**Table.** Response Rates in Immune Checkpoint Inhibitor Trials

Immune checkpoint inhibitor trial	Patients, No.	PMID (year published)	Age, median (IQR), y	Male sex, No. (%)	Follow-up, median (IQR), mo^a^	Survival, median (95% CI), mo		Response, No./total No. (%)	
						OS	PFS	Objective	Complete
First-line treatment									
Pembrolizumab plus chemotherapy: KEYNOTE 361	351	34051178 (2021)	69 (62-75)	272 (78)	31.7 (27.7-36.0)	17.0 (14.5-19.5)	8.3 (7.5-8.5)	192/351 (55)	53/351 (15)
Pembrolizumab: KEYNOTE 361	307	34051178 (2021)	68 (61-74)	228 (74)	31.7 (27.7-36.0)	15.6 (12.1-17.9)	NA	93/307 (30)	34/307 (11)
Pembrolizumab: KEYNOTE 052	370	32552471 (2020)	74 (34-94)	286 (77)	≥24 (minimum follow-up)	11.3 (9.7-13.1)	2.2 (2.1-3.4)	106/370 (29)	33/370 (9)
First-line maintenance treatment									
Avelumab: JAVELIN Bladder 100	350	32945632 (2020)	68 (37-90)	266 (76)	≥19 (NA)	21.4 (18.9-26.1)	3.7 (3.5-5.5)	34/350 (10)	21/350 (6)
Salvage treatment									
Pembrolizumab: KEYNOTE 045	270	31050707 (2019)	67 (29-88)	200 (74)	27.7 (NA)	10.1 (8.0-12.3)	2.1 (2.0-2.2)	57/270 (21)	25/270 (9)
Nivolumab: CheckMate 275	270	28131785 (2017)/32532789 (2020)	66 (38-90)	211 (78)	33.7 (minimum follow-up)	8.6 (6.1-11.3)	1.9 (1.9-2.3)	56/270 (21)	18/270 (7)
Avelumab: JAVELIN Solid Tumor	242	33037118 (2020)	69 (30-89)	178 (72)	31.9 (range, 24-43)	7.0 (5.9-8.5)	1.6 (1.4-2.7)	40/242 (17)	10/242 (4)

Abbreviations: NA, not applicable; OS, overall survival; PFS, progression-free survival; PMID, PubMed identifier number.

^a^ Follow-up times are reported in months as medians with range or IQR or as minimum follow-up time.

## Discussion

The use of ICIs significantly expanded over the past decade and has transformed the management of mUC. Current guidelines recommend the first-line use of pembrolizumab only for patients who are ineligible for platinum treatment, while avelumab is recommended as maintenance therapy for patients who did not have disease progression with first-line chemotherapy.^5^ Pembrolizumab, nivolumab, and avelumab have been FDA approved as salvage treatment of mUC for patients whose disease progressed while undergoing chemotherapy. When assessing long-term outcomes with ICIs for metastatic cancers using the American Society of Clinical Oncology value framework, only 3 of 24 FDA-approved drugs were notable for long-term survival benefits, but none of the 3 indications regarded patients with mUC.^6^ The significant decrease of patients at risk within the treatment groups of these trials seems to reflect a lack of durable survival benefit for most patients with mUC. Our study is limited by its reliance on published clinical trial data, and the decrease in number at risk likely reflects both a lack of long-term follow-up for later time points and survival. Although a small proportion of patients achieve long-term survival in the real-world setting, it remains difficult to identify such outliers a priori due to lack of robust predictive biomarkers with proven clinical utility. Novel therapeutic combinations (eg, antibody-drug conjugates or targeted therapies with ICIs and future biomarkers with clinical utility) may further improve outcomes for this disease. Our findings are hypothesis generating and need validation.

## Notes

Supplement.
Data Sharing Statement
