# Supplementary material for: Immune Checkpoint Inhibitors and Long-term Survival of Patients With Metastatic Urothelial Cancer
Source: JAMA Netw Open. 2023 Apr 12;6(4):e237444. doi: 10.1001/jamanetworkopen.2023.7444 (PMC10098944; doi:10.1001/jamanetworkopen.2023.7444)
Supplement: Supplement. — Data Sharing Statement [file jamanetwopen-e237444-s001.pdf]

Data Sharing Statement

Zhu. Immune Checkpoint Inhibitors and Long-term Survival of Patients With Metastatic Urothelial Cancer. *JAMA Netw Open*. Published April 12, 2023.
doi:10.1001/jamanetworkopen.2023.7444

Data
Data available: Yes
Data types: Data (not involving human participants)
How to access data: Data can be made available upon request. Please contact alz9028@nyp.org.
When available: With publication

Supporting Documents
Document types: None

Additional Information
Who can access the data: Anyone requesting the data
Types of analyses: For any purpose
Mechanisms of data availability: With investigator support
